# Supplementary material for: The experience of being a mother with end stage renal disease: A qualitative study of women receiving treatment at an ambulatory dialysis unit
Source: PLoS One. 2021 Sep 27;16(9):e0257691. doi: 10.1371/journal.pone.0257691 (PMC8476019; doi:10.1371/journal.pone.0257691)
Supplement: S1 Interview guide review — (DOCX) [file pone.0257691.s001.docx]

**INTERVIEW GUIDE**

| **THE FIRST STAGE OF DATA COLLECTION**  What is your experience with CKD and being a mother and taking care of your children? |
| --- |
| **THE SECOND STAGE DATA COLLECTION**   1. Do you consider that ESKD has changed your life? 2. How was it when you were diagnosed with ESKD? 3. What was the most relevant issue for you? 4. What challenges have you faced being a mother and/or caring for your children having ESKD?" 5. What are the most relevant changes that have taken place in your family life? 6. Do you think the disease has impacted your family, and your children?, If so, how? 7. Can you describe your emotional experience of being a mother and having ESKD in a single sentence?   Additionally, “Please tell me more about that”, was also used during all the interviews (if needed) to enhance the depth of the discussion of a specific topic. During the interview, at the women’s request, it was clarified that when they were asked about the care of their children during RRT, this included both the care provided to the infants and the daily care that the woman provided to the remaining children she already had. |
