# Supplementary material for: The experience of being a mother with end stage renal disease: A qualitative study of women receiving treatment at an ambulatory dialysis unit
Source: PLoS One. 2021 Sep 27;16(9):e0257691. doi: 10.1371/journal.pone.0257691 (PMC8476019; doi:10.1371/journal.pone.0257691)
Supplement: Attachment — Submitted filename: Response to Reviewers R2.doc [file pone.0257691.s003.doc]

**RESPONSE LETTER PONE-D-21-15320R1**

**Decision on Manuscript PONE-D-21-15320R1**

**Entitled**: **The experience of being a mother with end stage renal disease: A qualitative study of women receiving treatment at an ambulatory dialysis unit**

**Journal: *PLOS ONE***

We would like to thank the Editors and the Reviewers for their careful consideration of our manuscript. We would also like to thank the Reviewers’ suggestions, which we believe have enhanced the quality of the manuscript. We have highlighted (in yellow) all the changes we have made throughout the text. Below please find a detailed list of how we have addressed each comment.

**Reviewers' Comments to Author:**

**REVIEWER: 2**

**A few things:**

**I think this sentence in the abstract conclusion may still be misleading page 3 line 3-4:**

**'These findings are also relevant to4 women requiring long-term dialysis.' In fact these findings are ONLY relevant to women on dialysis. I would recommend removing the word 'also'. The findings are not relevant to women who have ESKD but are transplanted.**

**Response:** We have followed the reviewer´s recommendations and clarify the sentence.

Deciding to become a mother and taking care of children represents a challenge for women with ESKD, coupled with the losses in their lives caused by the disease. These findings are only relevant to women on dialysis.

**Page 3 line 13 - 14 - I still think that for the causes of CKD, autoimmune disease and genetic renal disease should be included in this list. I realise they are discussed later but they should be mentioned from the outs**

**Response:** We have followed the reviewer´s recommendations and included the causes of kidney disease.

A significant percentage of the population suffer from CKD, which is related to highly prevalent phenomena or diseases, such as ageing, arterial hypertension, diabetes, cardiovascular disease, autoimmune disease or genetic renal disease [3].

**Page 15 line 3 - typo - CDK should read CKD**

**Response:** We have followed the reviewer´s recommendation and change it.

Gender differences exist in kidney disease [10]. Women have a higher prevalence of specific autoimmune diseases that, together with pregnancy, form a unique challenge for women at risk of CKD and AKI [10].

**Recommend that 'ESRD' be changed to '****ESKD' and acute kidney disease by changed to AKI (acute kidney injury) throughout the manuscript in line with current nomenclature.**

**Response:** We have followed the reviewer´s recommendations and change it in the manuscript

The authors believe that the comments made by the reviewers have improved the quality of the manuscript. We hope that the changes made meet all the observations and expectations of the reviewers.

The Authors
